# Digital Holographic Microscopy, a Method for Detection of Microorganisms in Plume Samples from Enceladus and Other Icy Worlds

**DOI:** 10.1089/ast.2016.1616

**Published:** 2017-09-01

**Authors:** Manuel Bedrossian, Chris Lindensmith, Jay L. Nadeau

**Affiliations:** ^1^ Graduate Aerospace Laboratories (GALCIT) and Medical Engineering, California Institute of Technology, Pasadena, California.; ^2^ Jet Propulsion Laboratory, California Institute of Technology, Pasadena, California.

## Abstract

Detection of extant microbial life on Earth and elsewhere in the Solar System requires the ability to identify and enumerate micrometer-scale, essentially featureless cells. On Earth, bacteria are usually enumerated by culture plating or epifluorescence microscopy. Culture plates require long incubation times and can only count culturable strains, and epifluorescence microscopy requires extensive staining and concentration of the sample and instrumentation that is not readily miniaturized for space. Digital holographic microscopy (DHM) represents an alternative technique with no moving parts and higher throughput than traditional microscopy, making it potentially useful in space for detection of extant microorganisms provided that sufficient numbers of cells can be collected. Because sample collection is expected to be the limiting factor for space missions, especially to outer planets, it is important to quantify the limits of detection of any proposed technique for extant life detection. Here we use both laboratory and field samples to measure the limits of detection of an off-axis digital holographic microscope (DHM). A statistical model is used to estimate any instrument's probability of detection at various bacterial concentrations based on the optical performance characteristics of the instrument, as well as estimate the confidence interval of detection. This statistical model agrees well with the limit of detection of 10^3^ cells/mL that was found experimentally with laboratory samples. In environmental samples, active cells were immediately evident at concentrations of 10^4^ cells/mL. Published estimates of cell densities for Enceladus plumes yield up to 10^4^ cells/mL, which are well within the off-axis DHM's limits of detection to confidence intervals greater than or equal to 95%, assuming sufficient sample volumes can be collected. The quantitative phase imaging provided by DHM allowed minerals to be distinguished from cells. Off-axis DHM's ability for rapid low-level bacterial detection and counting shows its viability as a technique for detection of extant microbial life provided that the cells can be captured intact and delivered to the sample chamber in a sufficient volume of liquid for imaging. Key Words: *In situ* life detection—Extant microorganisms—Holographic microscopy—Ocean Worlds—Enceladus—Imaging. Astrobiology 17, 913–925.

## 1. Introduction

The icy moons of Jupiter and Saturn may be the most likely places in the Solar System for extraterrestrial life, and NASA now has as an explicit goal the search for signs of life on Europa (Prieto-Ballesteros *et al.,* 2011; Gleeson *et al.,* 2012), Enceladus, and Titan (Carr *et al.,* 2013; McKay *et al.,* 2014; Konstantinidis *et al.,* 2015; NASA, 2016). Such life is expected to be prokaryotic, anaerobic, and present at potentially very low densities. There is as yet no consensus in the astrobiology community as to the best method for detecting such life. Because no mission since Viking in 1976 has attempted to find extant life on another planet, there has been limited development of technologies and instruments for bacterial identification and counting during space missions (Hoover, 2015). Unambiguous signs of prokaryotic life can be difficult to obtain with *in situ* robotic instruments. Purely chemical means of detection may have difficulty distinguishing extant life from prebiotic organics or extinct life, and they do not show sizes, shapes, or activity of cells. Since one of the key features of life is local disequilibrium, some kind of cell-like structure is necessary (Georgiou and Deamer, 2014), so there is increasing interest in the use of direct imaging to detect extant cells on icy worlds (Nadeau *et al.,* 2016).

The Viking mission did not attempt to image cells; its approach to bacterial detection was based upon traditional methods of bacterial identification and enumeration, which involve culturing and plate counts. Such an approach was the gold standard at the time, but modern molecular biology techniques have shown that culturable cells represent <10% of total prokaryotes and require 1–2 weeks of incubation for maximum success. On another planet, with unknown nutritional requirements, culturing would be expected to be even less successful. Epifluorescence microscopy has largely replaced plate counts for rapid and accurate bacterial enumeration. Nucleic acid–labeling fluorescent dyes such as 4′,6-diamidino-2-phenylindole dihydrochloride (DAPI), SYBR Green, or acridine orange are the typical probes used (Marie *et al.,* 1997; Lebaron *et al.,* 1998; Noble and Fuhrman, 1998), though many studies use fluorescent indicators of Gram sign (Lauer *et al.,* 1981; Sizemore *et al.,* 1990; Mason *et al.,* 1998; Saida *et al.,* 1998; Forster *et al.,* 2002). These studies generally report limits of detection of ∼10^5^ cells/mL based upon visual inspection of slides. Improvements in limits of detection are possible with filtration. A liquid sample is vacuum-filtered onto a membrane, usually polycarbonate and ideally black to reduce background. Low-background dyes such as the DNA stains mentioned above may be applied directly to the filter (Bitton *et al.,* 1983). For accurate counting by eye, ∼10^5^ cells are required per filter, which means that, for samples more dilute than ∼10^5^ cells/mL, significant sample volumes must be collected and filtered. Laser-scanning devices have been developed in order to systematically explore all regions of the filter and thus reduce the number of cells required for counting, pushing limits of enumeration down to ∼10^2^ cells/mL (Broadaway *et al.,* 2003). However, these are not available in most laboratories and require sophisticated hardware as well as software that permits expert confirmation that observed particles are bacteria and not debris. Performing these concentration and staining steps robotically would present a significant challenge, even if a high-performance fluorescence microscope could be qualified for flight.

Alternatives to fluorescence microscopy that increase sample throughput and minimize moving parts have been sought for astrobiological applications. Digital holographic microscopy (DHM) represents one promising approach that has been demonstrated in aqueous environments on Earth (lakes and oceans) (Jericho *et al.,* 2010; Lindensmith *et al.,* 2016). A key advantage of DHM over traditional light microscopy is the simultaneous imaging of a thick sample volume at high resolution—for typical optics needed to image prokaryotes, no loss in resolution will be seen with samples as thick as a millimeter. This represents an approximately 100-fold improvement in depth of field over high-power light microscopy. An additional advantage of DHM is its ability to monitor both intensity and phase of the images, so that organisms that are nearly transparent in intensity (such as most biological cells) appear clearly in phase (Cuche *et al.,* 2000; Kemper *et al.,* 2007). As it is a label-free technique, no dyes are used. This is an advantage for possible spaceflight applications, since dyes may not survive the conditions of a mission and—more importantly—cannot be assumed to work with possible extraterrestrial organisms, which may not use DNA or RNA for encoding.

In this paper, we demonstrate a limit of detection of 10^3^ cells/mL using a custom off-axis digital holographic microscope (DHM) specifically designed for field and astrobiology applications (Wallace *et al.,* 2015a). The instrument has no moving parts, and object and reference beams are mounted along a common path so that they cannot be misaligned by mechanical shock. It has been demonstrated in the field and is currently at technology readiness level (TRL) 5 (Lindensmith *et al.,* 2016). The limit of detection was obtained theoretically and experimentally by sequentially sampling a number of sample volumes. The utility of the theoretical model for determining microscopic limits of detection has been demonstrated, showing it to be a useful tool to select the number of samples or fields of view that must be examined. The ability to unambiguously identify living microorganisms from oligotrophic environments was then explored in two samples from the Canadian High Arctic that represent different bacterial populations, concentrations, and levels of activity. We were able to clearly identify life in one of the samples, a cold spring pool harboring ∼10^4^ cells/mL, without preconcentration or any other steps. In a sample of glacier ice, very few active organisms were seen immediately after thawing, but motility was greatly enhanced by overnight incubation in enriched medium. Concentration was necessary to observe multiple cells per frame in this highly oligotrophic sample.

Label-free techniques suffer from potential lack of specificity. When organisms are motile or possess resolvable subcellular features, they are clearly alive. However, nonmotile, featureless, micron-sized prokaryotes are difficult to distinguish from mineral grains or other inorganic material when using traditional light microscopy. This is one of the primary criticisms of the use of microscopy for life detection. Simply increasing spatial resolution is not the answer; bacteria can look ambiguous even under electron microscopy, as exemplified by the ALH84001 meteorite (Thomas-Keprta *et al.,* 2002). The context in which cell-like objects appear is important to establish the presence of multiple identically shaped and sized, highly regular features that may suggest life, so obtaining large fields of view at micron resolution is highly desirable. The challenge is to develop label-free techniques that distinguish organic from inorganic material. Both of our Arctic samples contained structures ranging from 1 to 50 μm in size. Although the sharp edges of the larger objects suggested that they were crystals, they remained ambiguous under intensity imaging. Using quantitative phase reconstructions of the holograms, we were able to estimate the index of refraction of both microorganisms and crystals. Such quantitative phase imaging is an emerging field in biology, with reported capability to resolve refractive index changes as small as Δ*n* = 10^−4^ (Cuche *et al.,* 2000; Indebetouw *et al.,* 2006; Kemper *et al.,* 2007). We found that microbial cells had indices of refraction that differed from water only in the second decimal place (1.37 as opposed to 1.33). On the other hand, crystal indices ranged from 1.5 to 2.0 at 405 nm illumination, making them clearly distinguishable from cells and suggesting insights into their chemical composition.

These studies help define the minimum sample volume, cell concentration, and cell size that would be necessary for detection of extant life in an extraterrestrial sample. The challenge on icy worlds is to collect sufficient sample and deliver the cells intact to the instrument. A flyby through the heart of the Enceladus plume at an altitude of 50 km is expected to collect on average ∼1 × 10^−4^ mL of H_2_O on a 20 × 20 cm^2^ detector (Porco *et al.,* 2017, in this issue). For a bulk microbial load of 10^4^ cells/mL, which is a representative concentration in the upper ocean layer at the bottom of the ice shell based on geothermal energy assumptions, as well as an analysis of plume images of Enceladus (Porco *et al.,* 2017, in this issue), this would yield ∼1 cell per transect at an altitude of 50 km. Bubble scrubbing could improve this number by factors up to the thousands (Porco *et al.,* 2017, in this issue). Multiple flybys and collection of the entire sample into a small volume would augment the detection limits of DHM. Alternatively, Enceladus orbiters executing repeated fly-throughs of the plume, penetrators dropped from orbit, or landers could collect larger sample volumes. In the case of Enceladus, nondestructive capture of microorganisms would be possible at orbital altitudes of tens of kilometers, and ∼96% of its plume particles would fall back to the surface, making nondestructive capture on the surface possible as well. Future laboratory work is necessary to simulate Enceladus capture and to define the parameters of a mission needed for imaging extant life. We conclude this paper with a discussion of these experiments.

## 2. Experimental Methods

The instrument used throughout this experiment was an off-axis DHM developed specifically for imaging prokaryotes (Wallace *et al.,* 2015b). The optical specifications of this instrument are given in Table 1, along with properties of a conventional light microscope having similar spatial resolution and field of view.

**Table 1.** Specifications of the Off-Axis DHM Instrument Used and Comparable Brightfield Microscope (“Conventional”)

*Property [unit]*	*Value (DHM)*	*Value (Conventional)*
Illumination wavelength [nm]	405	White light
Objective numerical aperture [-]	0.3	0.4
Magnification [-]	20	20
Field of view [μm × μm]	360 × 360	400 × 400
Depth of field [μm]	800	6
Lateral resolution [μm]	<1	<1

### 2.1. Hologram reconstructions and quantitative phase imaging

Amplitude and phase reconstructions were performed with the software Koala (LynceeTec), which uses a preconditioned conjugate gradient phase unwrapping algorithm (Kaufmann *et al.,* 1998; Yong and Bryan, 2009). Thickness was calculated with the Local Thickness plug-in for Fiji (Schindelin *et al.,* 2012), which defines local thickness as the diameter of the largest sphere that fits inside the object and contains the point; the algorithms were described in depth by Hildebrand and Rüesgsegger (1996). The local thickness algorithm was run on a z-projection of 200 amplitude stacks reconstructed every 1.2 μm in depth.

### 2.2. Experimental limits of detection

The test bacteria used for the quantification of the limits of detection was *Bacillus subtilis* ATCC6051 (American Type Culture Collection), which was grown overnight at a temperature of 30°C in lysogeny broth (LB, Becton Dickinson) to an optical density of OD_600_ = 0.8. *Bacillus subtilis* was chosen as the test strain due to its easily recognizable shape and swimming pattern, which prevents false-positive readings resulting from debris or common environmental contaminants.

From the overnight culture, a total of eight dilutions were prepared of 10^*n*^ (LB to bacterial culture) where *n* = 1, 2, 3, … 8. A Petroff-Hauser counting chamber (Electron Microscopy Sciences) was used to determine the correspondence between optical density and number of cells. Cells were placed into the chamber, and 25 regions were counted and averaged on a Nikon phase contrast microscope with a 63 × air objective. This gave a value of 10^8^ cells/mL at OD = 0.8. Thus, the test concentrations were 10^*m*^ cells/mL, where *m* = 0, 1, 2, … 8.

A total of nine sample chambers were prepared, one for each concentration, to avoid any possibility of contamination from one concentration to another. Each concentration was loaded into the sample chamber with a sterile syringe, with sterile-filtered dH_2_O loaded into the reference channel of the sample chamber. Three sample volumes were imaged per concentration. The number of imaged sample volumes was limited to avoid excessive data sizes, while providing a high probability of bacterial detection. Concentrations of 10^2^ and 10^3^ cells/mL were imaged over a total of 10 sample volumes in order to validate the theoretical limit of detection further than a single experiment.

For each sample volume, holograms were acquired at 6.67 frames per second (fps) for 20 s at a time. Once acquired, the holograms were numerically reconstructed in both phase and intensity at various focal planes through the 800 μm thick sample.

With the data sets numerically reconstructed throughout the depth of the sample in both phase and intensity, the detection limits of the DHM instrument were quantified. Due to the broad range in bacterial concentrations that were analyzed, it is possible to determine the DHM's lower limit of detection as well as its upper limit of detection.

The DHM's lower limit of detection was determined by the analysis of all focal planes by the human eye. Median background subtraction, a de-noising algorithm, was used to aid in the detection process. Each focal plane for each data set was analyzed in both phase and intensity reconstructions until the presence of bacteria was seen. All concentrations were also analyzed through the median subtraction of raw holograms. Numerical reconstruction is a process with high computational overhead; thus bacterial detection in raw holograms is very desirable for the practical implementation of DHM as an accurate bacterial counting method.

The upper limit of detection was defined as the bacterial concentration at which the sample is so densely populated, the signal-to-noise ratio (SNR) of the bacterium is attenuated by 3 dB from its nominal value (∼30% reduction). For each data set, the lateral position of bacteria was recorded with a MATLAB routine. This routine used those bacterial coordinates to cycle through all reconstructed focal planes in order to calculate the SNR to find that bacterium's focus plane. The in-focus SNRs of the bacteria were used to compare the average SNRs at various concentrations to determine the upper concentration limit.

### 2.3. Environmental samples

Two environmental samples were examined by DHM and fluorescence microscopy. The first was a sample from the main pool of a cold spring at Gypsum Hill, Axel Heiberg Island, Nunavut, Canada (longitude −90°43′05″, latitude 79°24′30″). The cold springs of Gypsum Hill contain large masses of biofilms that collect into “streamers” in the outflow channels of the springs; the dominant organism in these biofilms is the sulfur reducer *Thiomicrospira*. The main pools contain <10^4^ organisms/mL (Niederberger *et al.,* 2009). The sample was collected by Lyle Whyte of McGill University in March 2016 and shipped on ice (unfrozen), where it was stored at +4°C. The second sample was a piece of englacial ice from White Glacier, Axel Heiberg Island, Nunavut, Canada (longitude −90°50′, latitude 79°30′), collected by Lyle Whyte of McGill University in March of 2016 and shipped frozen. The sample was thawed at +4°C in a sterile hood, and observations were made immediately.

Digital holographic microscopy was performed on the samples in a 0.8 mm deep imaging well (Electron Microscopy Sciences) without preconcentration or staining. If nothing was observed, the sample was concentrated 10- to 100-fold by syringe filtration through a 0.22 μm filter. If cell-like objects but no activity was observed, samples were treated with 1 m*M* serine or one-half strength 2216 Marine broth (Difco) and imaged immediately, after 1 h, and after overnight incubation at +4°C and room temperature.

Staining for fluorescence microscopy was performed with the Live/Dead BacLight Bacterial Viability kit (Molecular Probes). Dye was added to samples concentrated 10–100 × and incubated on a rocker for 30 min. Slides were prepared with SloFade Gold anti-photobleaching mounting medium (Molecular Probes) and examined on an Olympus IX71 inverted fluorescence microscope with Hg lamp illumination and an Endow GFP filter used to visualize the “Live” stain (SYTO9), and a Texas Red filter used to visualize the “Dead” stain (propidium iodide). Images were captured in grayscale on an Andor Zyla CMOS camera and pseudo-colored. Images of Gypsum Hill biofilms under confocal and environmental scanning electron microscopy were taken previously with the methods described by Clarke *et al.* (2010).

## 3. Results

### 3.1. Theoretical limits of detection of microscopic imaging

A statistical model based on Bernoulli statistics was used to derive an expression for the theoretical limits of detection of any microscopy instrument based on its optical characteristics. The general assumption for this model is that the bacterium need only be in the field of view (FOV) of the instrument to be detected. Most biological samples are transparent, but due to developments in label-free imaging techniques such as phase contrast microscopy, it is possible to image them without dyes or stains, thus making this a valid assumption.

Assuming that the bacterial sample is thoroughly mixed such that the bacterial concentration is uniform throughout, the probability that a single bacterium will be in the FOV of the image is given by $p = {V_{{ \rho _{ \rm{b}}}}}$, where *V* is the sample volume (FOV × depth of field) in milliliters (always much smaller than 1) and *ρ*_b_ is the bacterial concentration of the sample in cells per milliliter.

Applying the assumption of Bernoulli statistics, the law of total probability states *p* + *p*_not_ = 1, where *p*_not_ is the probability that a bacterium is *not* in the FOV of the instrument.

If this sampling is repeated *x* times, the law of total probability becomes ${p_{{ \rm{eff}}}} + p_{{ \rm{not}}}^x = 1$, where *p*_eff_ is the effective probability that at least one bacterium will be imaged after *x* samples. This results in an expression that estimates the number of sample volumes to image in order to detect bacteria as a function of concentration at a given confidence level (*p*_eff_).

\begin{align*}
x = { \frac { \ln \left( { 1 - { p_ { { \rm { eff } } } } } \right) }  { \ln \left( { 1 - { V_ { { \rho _ { \rm { b } } } } } } \right) } } \tag { 1 } 
\end{align*}

A specific advantage of the off-axis DHM used is the fact that it contains two channels that are recorded simultaneously, which requires two arms of light to create interference patterns or fringes. For sparse samples, it is possible to load sample in both channels as opposed to just one, which effectively doubles the sample volume capability of the instrument. Imaging done with an off-axis DHM with sample in both channels of the sample chamber is referred to as “Detection Mode” throughout the scope of this document.

**FIG. 1.**
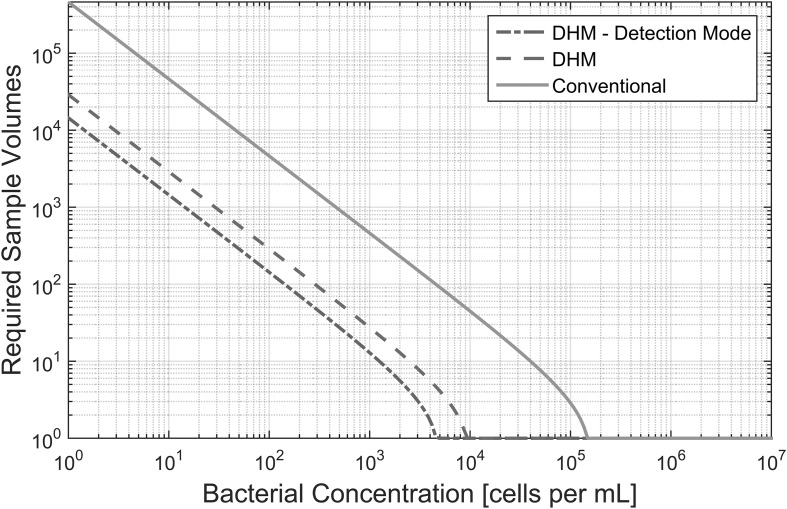
Number of required sample volumes to detect bacteria at various concentrations with a confidence level of 95%.

Figure 1 plots the number of sample volumes that must be imaged as a function of bacterial concentration in order to have a detection confidence level of 95% (*p*_eff_ = 0.95 in Eq. 1). In the figure, the same calculation is shown for a conventional light microscope (parameters in Table 1).

Table 2 shows the number of sample volumes that must be imaged when using off-axis DHM as well as a conventional light microscope in order to have a detection confidence level of 50%. This shows the minimum number of samples that must be imaged in order to have a sufficiently large possibility of detecting bacteria.

**Table 2.** Minimum Number of Required Sample Volumes to Detect Bacteria (Detection Confidence of 50%)

*Concentration [cells/mL]*	*DHM*	*Conventional*
10^0^	6,686	106,967
10^1^	669	10,697
10^2^	67	1,070
10^3^	7	107
10^4^	1	11
10^5^	1	1

The aforementioned analysis assumes the FOV to be instantaneous and constant; however, the ability to acquire image sequences allows for larger FOVs to be imaged. The increase in the instantaneous FOV to an *effective* FOV is proportional to the length of the image sequence recorded as well as the level of motility of the sample. Because this is difficult to quantify in a general case for such samples as bacteria, only a simple estimation is discussed here.

For the purposes of estimating the effective FOV, bacterial motility can be modeled as a one-dimensional random walk where each step of a bacterium has an equal probability of being forward or backward. Assuming that a bacterium takes a “step” every second, the net distance traveled by a bacterium can be expressed as

\begin{align*}
d = v \mathop \sum \limits_{i = 1}^k {{S_i}} \tag{2}
\end{align*}

where *v* is the average swimming speed of a bacterium in micrometers per second, and *S_i_* ∈{−1,1} corresponding to a step backward or forward over a total *k* steps. From the possible values of *S_i_*, the expected value of any $S_n^2$ is $E \left( {S_n^2} \right) = 1$.

Because the bacterium has an equal probability of going forward or backward, lim_*k*→∞_***d*** = 0, but for a *finite* number of steps, the magnitude of the net distance traveled by the bacterium is

\begin{align*}
\begin{split} \left\vert d \right\vert & = \sqrt {{d^2}} = \sqrt { \left( {v \sum \nolimits_{j = 1}^k {{S_j}} } \right) \left( {v \sum \nolimits_{i = 1}^k {{S_i}} } \right) } \\ & { \rm{ }} = \sqrt {{v^2} \sum \nolimits_{i = 1}^k { \sum \nolimits_{j = 1}^k {{S_i}{S_j}} } } = v \sqrt { \sum \nolimits_{i = 1}^k { \sum \nolimits_{j = 1}^k {{S_i}{S_j}} } } { \rm{ }} \\ &= v \sqrt k \\\end{split}
  \tag{3}\end{align*}

resulting in a net distance on the order of |***d***| = $v \sqrt k$ over *k* seconds. For any given sample chamber, bacteria are only free to move in and out of the FOV of the instrument across four boundaries of the sample volume because the other two are bounded by glass. Thus, the effective sample volume as a function of image sequence duration (*t*) is ${V_{{ \rm{eff}}}} = V + 2zv \sqrt t \left( {x + y} \right)$, where *x, y,* and *z* are the length, width, and height of the sample volume, respectively.

### 3.2. Lower limit of detection

The results from the analysis of the data collected with off-axis DHM at various bacterial concentrations are shown in Table 3. Because of the known swimming behaviors of *B. subtilis* (Ito *et al.,* 2005), the estimated probability of detection at each concentration includes the effective FOV approximation.

**Table 3.** Detection Analysis with Off-Axis DHM at Various Bacterial Concentrations, with Predicted Detection Probabilities at Each Concentration

	*Sample volume*			
*Concentration [cells/mL]*	*1*	*2*	*3*	*Probability of detection*^a^
10^0^	×	×	×	0.06%
10^1^	×	×	×	0.64%
10^2^	×	×	×	6.15%
10^3^	×	^*^	×	51.7%
10^4^	✓	✓	✓	100%
10^5^	✓	✓	✓	100%
10^6^	✓	✓	✓	100%
10^7^	✓	✓	✓	100%
10^8^	✓	✓	✓	100%

^a^ Predicted probabilities calculated using an *effective* FOV approximation.

× No bacteria were detected throughout the entire sample volume.

^*^ A single bacterium was detected in the phase reconstruction and hologram after median subtraction.

✓ Unambiguous presence and motility of bacteria even without median subtraction.

The results of imaging the samples with concentrations of 10^2^ and 10^3^ cells/mL, for a total of 10 sample volumes each, are shown in Table 4. The effective FOV approximation was used. The probabilities reported in Table 4 are the probability of detecting a single bacterium within the 10 sample volumes recorded. At a concentration of 10^3^ cells/mL, a total of three bacteria were seen. The probability of this occurring was estimated at 55.4%. This calculation was done assuming that each imaged sample volume was independent.

**Table 4.** Probability of Detection of Low Concentrations in 10 Sample Volumes

	*Sample volume*										
*Concentration [cells/mL]*	*1*	*2*	*3*	*4*	*5*	*6*	*7*	*8*	*9*	*10*	*Probability of detection*^a^
10^2^	×	×	×	×	×	×	×	×	×	×	25.7%
10^3^	×	×	×	✓	×	×	×	✓	✓	×	91.6%

^a^ Predicted probabilities calculated using an *effective* FOV approximation.

× No bacteria were detected throughout the entire sample volume.

✓ Unambiguous presence and motility of bacteria even without median subtraction.

### 3.3. Upper limit of detection

**FIG. 2.**
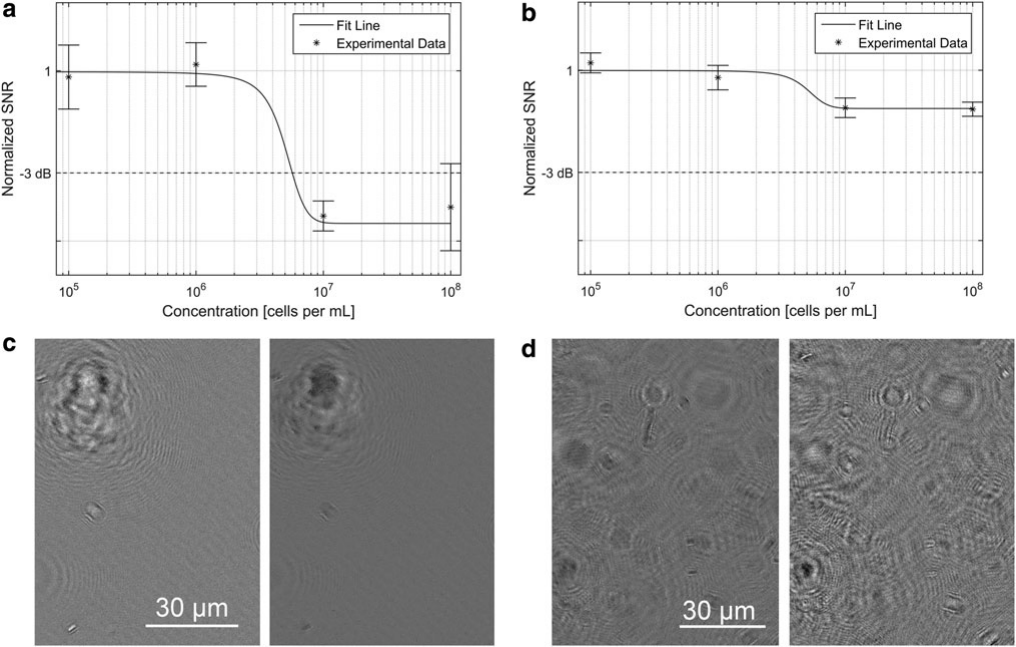
SNR as a function of bacterial concentration. (**a**) Normalized SNR as a function of bacterial concentration in amplitude images. The line is a smooth fit to the experimental data. (**b**) Normalized SNR as a function of bacterial concentration in phase images. The line is a smooth fit to the experimental data. (**c**) Amplitude images at 10^5^ cells/mL (left) and 6 × 10^6^ cells/mL (right). Note the overlapping Airy rings in the right panel. (**d**) Phase images at 10^5^ cells/mL (left) and 6 × 10^6^ cells/mL (right).

The SNR of intensity and phase reconstructions as a function of bacterial concentration is shown in Fig. 2a and 2b, respectively. At a concentration of ∼6 × 10^6^ cells/mL of *B. subtilis,* the signal to noise showed an abrupt drop resulting from multiple scattering events from the cells that impeded hologram reconstruction. This drop corresponded to the samples appearing “crowded,” with Airy rings from out-of-focus cells overlapping the in-focus cells (Fig. 2c, 2d). Although the signal to noise was reduced, reconstructions could still be performed with no further loss of quality until ∼10^8^ cells/mL. Above this point, hologram reconstructions were not meaningful.

## 4. Results: Environmental Samples

### 4.1. Gypsum Hill

**FIG. 3.**
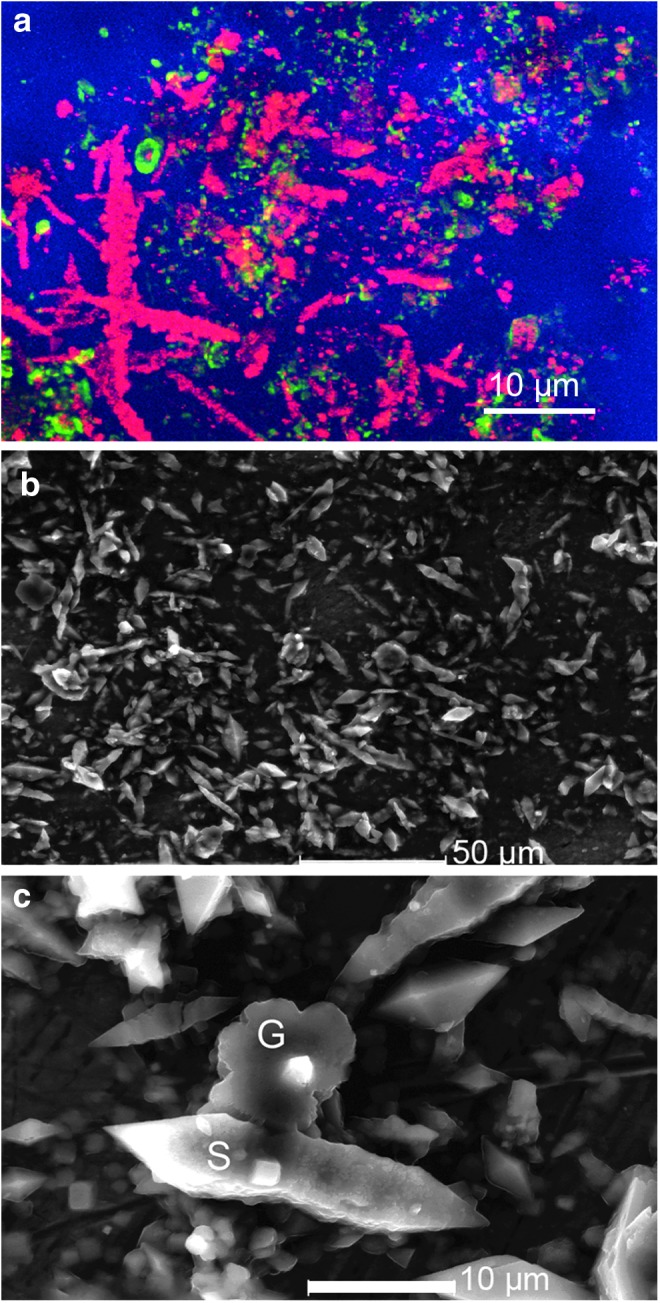
Confocal and electron microscopy of Gypsum Hill biofilms. (**a**) Confocal image in reflectance (red) and fluorescence (green). The reflectance image shows numerous spindle-shaped objects 5–20 μm in size. The green fluorescence results from bacterial labeling with the DNA stain SYTO BC (“Live”). (**b**) Environmental scanning electron microscopic image showing a variety of minerals, many of similar appearance to the spindle-shaped structures in (a). (**c**) Close-up of two minerals with small spot-size EDS analysis reveals gypsum (G) and elemental sulfur (S).

We have performed several previous studies of the microbiology of the Gypsum Hill cold springs (Nadeau *et al.,* 2008; Niederberger *et al.,* 2009; Pollard *et al.,* 2009; Rogers *et al.,* 2010). These cold springs possess dense biofilms in their outflow channels, whereas the main pool contains a very low concentration of microorganisms, below the limit of detection of conventional light microscopy. The organisms are too small to be imaged with a commercial in-line DHM (Jericho *et al.,* 2010), a result which led directly to the design of the instrument used in this paper. The biofilms consist almost entirely of sulfur-reducing bacteria of the genus *Thiomicrospira,* along with a variety of minerals (gypsum, sulfur, and halite). In one study, we reported a “real life-detection problem” because of the presence of ∼100 μm elongated features within the biofilm that stained ambiguously with Live/Dead and several other fluorescent stains, including acridine orange. Electron microscopy with energy-dispersive X-ray spectroscopy (EDS) was required to determine that these were crystals of elemental sulfur rather than cells, though they may have had fragments of bacterial cells on their surfaces (Rogers *et al.,* 2010). Fluorescence and electron microscopic images of fragments of streamer biofilm are shown in Fig. 3.

**FIG. 4.**
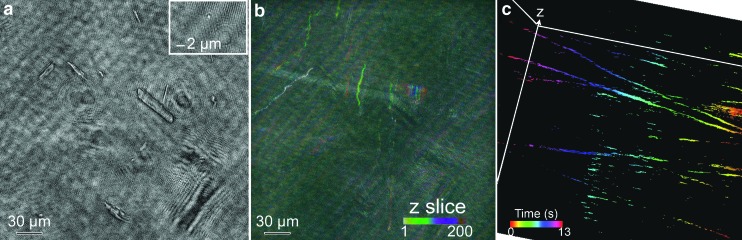
DHM amplitude reconstructions of images of Gypsum Hill cold spring water. (**a**) Single plane, full FOV showing multiple crystals. The inset shows a close-up of a bacterial cell that was identified as such by its motility. (**b**) Depth-coded maximum-intensity time projections of trajectories of organisms in the volume of view. (**c**) 3-D view of time-coded trajectories over 13 s of recording showing organisms on multiple z-planes.

Digital holographic microscopy examination of water from the main cold spring pool clearly showed the presence of the spindle-shaped sulfur crystals (Fig. 4a). Individual *Thiomicrospira* cells were very small relative to the FOV but could be readily identified by their swimming (Fig. 4b, Supplementary Video 1; Supplementary Data are available online at www.liebertonline.com/ast). Cells were present on multiple z-planes throughout the volume of view. While usually one cell, at most, was in sharp focus on a single z-plane, projections or 3-D reconstructions revealed a concentration of multiple motile organisms per imaged volume. Inspections of 10 video clips yielded an average of 5 ± 2 cells/volume for imaged volumes of 0.1 μL, or an average of 5 ± 2 × 10^4^ cells/mL. The majority of cells were motile; ambiguous cell-like objects without motility were rare.

### 4.2. White Glacier

In freshly melted, unprocessed samples from White Glacier, no cells were apparent in several minutes of observation under the DHM. The sample was then concentrated through a 0.2 μm filter. Concentrated samples were used to obtain direct cell counts using Live/Dead stain under conventional fluorescence microscopy. Results showed 1500–5000 cells/mL across five samples (not shown).

**FIG. 5.**
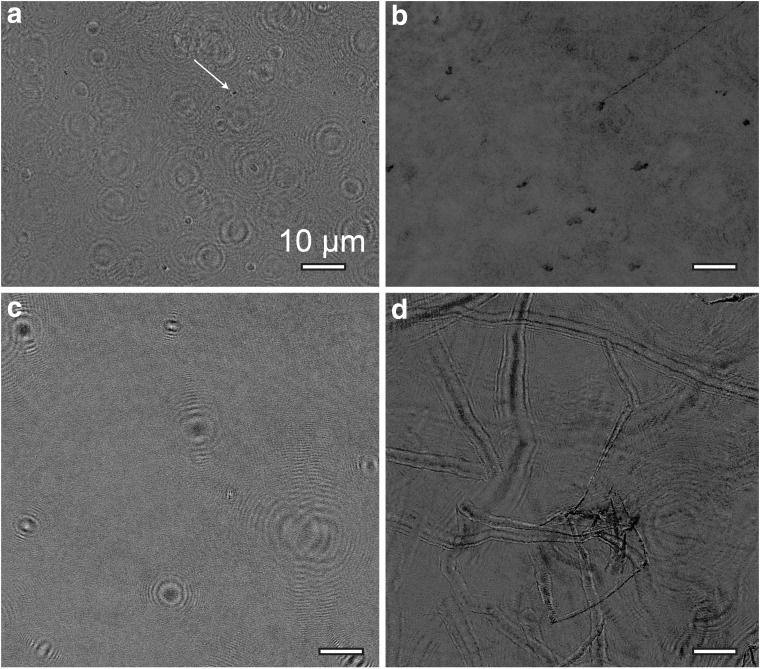
Glacier ice before and after medium supplementation. Images are median-subtracted amplitude reconstructions on a single plane. The samples were concentrated 50-fold. (**a**) Immediately after thawing, a large number of micrometer-scale objects were apparent, but only one (arrow) was motile. (**b**) Minimum-intensity projection over 30 s showing motility of one particle and Brownian motion of others. (**c**) After overnight incubation in one-half 2216 marine broth. Median subtraction removed all background particles; all objects seen were highly motile. (**d**) Minimum-intensity projection over 60 s showing motility of all objects in the field. The traces become thicker and thinner as the cells travel in and out of the selected focal plane.

At 50-fold concentration, the DHM FOV was relatively crowded with micron-sized particles (Fig. 5a). Most of the particles demonstrated Brownian motion, with <5% showing active swimming (Fig. 5b, Supplementary Video 2). Determining whether these particles were live but nonmotile cells or nonliving particles was performed in two ways. First, it was seen that overnight incubation of the glacier ice in one-half 2216 marine broth resulted in a dramatic increase in cell motility, along with increased ease in separating cells from their background. Nonmotile particles clustered near the bottom of the chamber and could easily be removed by median subtraction, yielding clean images of cells on multiple focal planes in both amplitude and phase (Fig. 5c, Supplementary Videos 3 and 4). Projection of trajectories over time showed the zigzag motility pattern characteristic of marine bacteria (Fig. 5d, Supplementary Video 5). Incubation with 1 or 10 m*M* serine or with glucose did not significantly increase motility (not shown).

### 4.3. Distinguishing cells from minerals using quantitative phase information

Phase images could be used to determine whether particles were nonmotile cells or mineral particles in both the White Glacier and Gypsum Hill samples by calculating their refractive index. The phase shift $\Delta \phi$ generated by an object of thickness *t* is related to the wavelength of illumination, λ, and the index difference between the object and the medium, Δ*n*, by (Rappaz *et al.,* 2005)

\begin{align*}
 { \frac { \Delta \phi }  { 2 \pi } } = \frac { { t \Delta n } }  { \lambda } \tag { 4 } 
\end{align*}

**FIG. 6.**
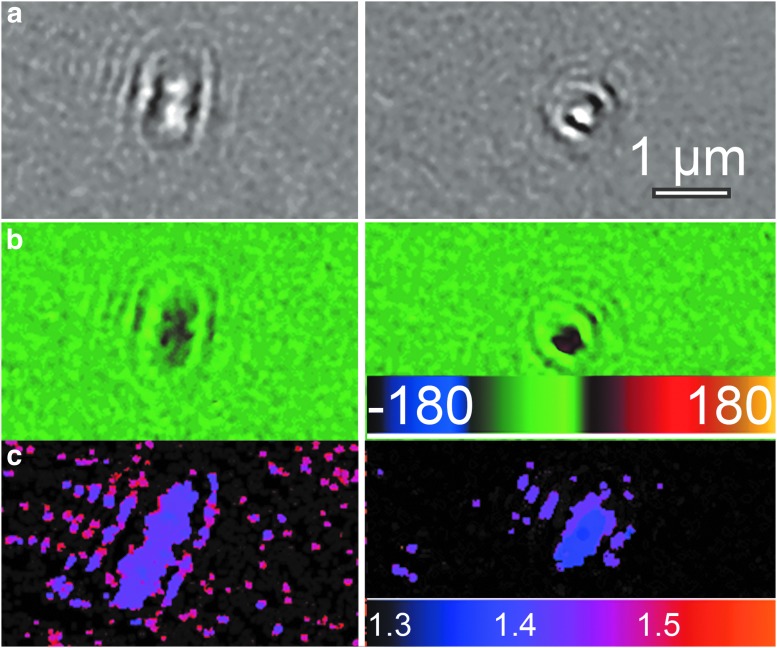
Phase and refractive index changes in motile cells. (**a**) A tumbling cell seen in amplitude. (**b**) The same cell in phase showing an increase in phase shift as the cell turns end-on. (**c**) Calculated index of refraction using the thickness measured from the amplitude images.

If $\Delta \phi$ > 2π, only the value modulo 2π is measured, with a discontinuity or “wrap” in the phase image. However, the bacterial cells were all so small and of such low refractive index that wrapping did not occur. Even if wrapping occurs, it can be undone provided that the distance over which the wrapping occurs is a few resolution elements. This is generally the case for low-index objects. Because the cells turned in all directions due to drift and/or motility, their thickness along all axes could be readily quantified from the intensity images. Figure 6 shows a cell changing direction with resulting increases in $\Delta \phi$ as it turns end-on and so becomes effectively thicker (Fig. 6a, 6b). The calculated refractive index was Δ*n* = 0.04, or adding the index of water as a background, *n* = 1.37 (Fig. 6c). This is in excellent agreement with values obtained for bacteria when using other methods (Balaev *et al.,* 2002; Liu *et al.,* 2014).

**FIG. 7.**
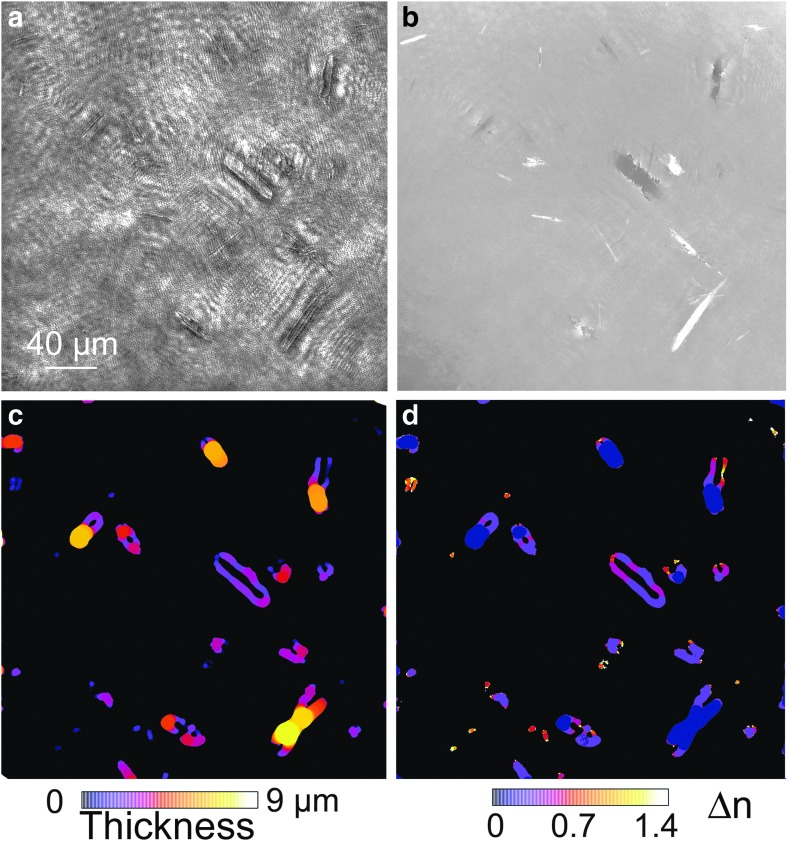
Determination of refractive index of minerals by local thickness estimation and quantitative phase imaging. (**a**) Intensity image. (**b**) Unwrapped phase image; the scale is −15 π to +15 π. (**c**) Thickness in micrometers determined from local thickness algorithm. (**d**) Index change over background (water, 1.33) as calculated from Eq. 4.

Because the minerals were irregular in shape and did not appear end-on, their exact thickness was more difficult to measure from the images than in the case of the bacteria. Nevertheless, sufficient information could be obtained to determine that the index of refraction of the crystals was too high to be consistent with cells, and demonstrate that all the minerals in the FOV had very similar refractive indices, suggesting that they were made of the same material. A local-thickness algorithm was applied to amplitude images of a Gypsum Hill sample containing many minerals (Fig. 7a), all of which showed large phase shifts (Fig. 7b). The thickness results obtained from the 3-D intensity stack were consistent with the spindle shapes suggested by electron microscopy; that is, the minerals were about as thick as they were wide (Fig. 7c). The index of refraction of the elongated minerals differed from background by ∼0.6–0.7, or an index of ∼1.9–2.0. This is consistent with elemental sulfur at 405 nm (Fig. 7d) (Samukawa *et al.,* 1992).

## 5. Discussion

The experimental results obtained correlate very well to the theoretical limits of detection derived in this work. According to Eq. 1, the probability of imaging a single bacterium with the DHM at a concentration of 10^3^ cells/mL after three sample volumes is 27.9%. Because the test strain, *B. subtilis,* has known swimming characteristics, accounting for the *effective* FOV after acquiring a 20 s image sequence results in a detection probability of 51.7%, according to Eqs. 1 and 3. The single bacterium detected at this concentration entered the FOV after the data acquisition sequence had begun. This, along with the fact that accounting for an effective FOV raised the detection probability above 50%, highlights the value of DHM's ability to not only image in three spatial dimensions but to do so in real time as well. The detection of this bacterium was made in both the phase reconstructions and raw holograms, both after median subtraction. The ease of detection of bacteria in the raw hologram shows its ability to be used for rapid, low-level detection and counting of bacteria. The bacterium was later seen in the intensity reconstructions but only after it was known to be there. This is due to the higher average SNR in phase than intensity reconstructions. As seen in Table 2, all higher concentrations yielded the unambiguous presence of bacteria in the raw holograms as well as phase and intensity reconstructions.

In quantifying the upper limit of detection of off-axis digital holography, the value of its phase imaging ability was further exemplified. We defined the upper limit of detection as the point where the average SNR attenuates by 3dB from its nominal value. In intensity reconstructions, this cutoff concentration is reached at 6 × 10^6^ cells/mL. In phase reconstructions, however, a drop in SNR is seen at the same concentration, but the SNR never attenuates by more than 1.5 dB, thus never crossing the cutoff threshold. In both intensity and phase reconstructions, the nominal SNRs were sufficiently high so that even after attenuation at 6 × 10^6^ cells/mL, the SNR still remained greater than unity. We have previously reported upper limits of ∼10^8^ cells/mL (Kuhn *et al.,* 2014), which reflects the point at which holograms can no longer be reconstructed because of the multiple scattering events. This upper limit remains valid in the studies performed here.

The combination of these two experiments shows that the high throughput capability of off-axis DHM provides an imaging method with a high-fidelity detection bandwidth of 1 × 10^3^ to 6 × 10^6^ cells/mL. This was confirmed by examining samples from highly oligotrophic environments. In samples containing ∼10^4^ cells/mL where a majority were motile, an active cell could be expected in any given 30 s video recording. In samples containing ∼10^3^ cells/mL, of which a majority were nonmotile, several 30 s videos were required to obtain a single active cell. Increasing cell concentration by simple filtration, as well as increasing the fraction of motile cells by supplementing the medium, increased the probability of detection and yielded multiple cell trajectories per FOV. The lower limit of detection can be effectively extended by using continuous flow of source material through the sample volume. Because the lower limit is improved linearly with the number of samples (and thus linearly in time), significant improvements can be gained in modest times; it is reasonable to survey a full milliliter of material over the course of an hour. Such surveys would benefit from improved algorithms for the autonomous detection of objects and motility in the FOV. The effects of steady flow can be subtracted to distinguish the effects of flow from motility.

Label-free light microscopy often suffers from the inability to distinguish living cells from similarly shaped mineral grains or microparticles (Rogers *et al.,* 2010). However, the quantitative phase imaging provided by DHM permits calculating the index of refraction of particles, which can clearly distinguish between cells (which have an index near that of water) and minerals (which have a much higher refractive index). In the Gypsum Hill samples, in particular, most mineral grains had a very high index of refraction: 2.0, corresponding to elemental sulfur. For life-detection purposes, multiple approaches are possible to enable index measurements. One simple approach may be to include beads or features of known size and refractive index in sample chambers, so that particles of similar size may be compared in phase images. On Enceladus, it is known that there are silica particles (Hsu *et al.,* 2015), with a refractive index of 1.46 at 405 nm. Including similar grains as a reference point for refractive index may be of value. Any mineral grain will have an index significantly greater than that of H_2_O. Conversely, imaging of a cell-like object with a refractive index very close to that of H_2_O suggests something that is at least an aqueous vesicle, and possibly alive. Detection of complex organic chemistry via other means (*e.g.,* by feeding the sample into a mass spectrometer) would confirm that the cell-like objects colocalized with biosignature molecules.

A multiwavelength flight instrument would be ideal for dispelling any ambiguity, as the multiple wavelengths allow for elimination of *t* in Eq. 4 if the mineral's refractive index changes significantly with wavelength (Rappaz *et al.,* 2005, 2008; Parshall and Kim 2006; Khmaladze *et al.,* 2011). This is true of sulfur (Samukawa *et al.,* 1992) but not of gypsum (Roush *et al.,* 2007). However, multiwavelength DHM is relatively complicated to implement and analyze because of chromatic aberrations and location of the images on different focal planes. Development of such an instrument for flight would require some development since such instruments are currently at low technology readiness levels.

One of the key questions for Enceladus is whether enough material could be captured from a plume flyby to permit direct imaging of cells. If all the cells from 10 flybys could be aggregated into a microliter of liquid, this would yield a sample at ∼1 × 10^4^ cells/mL, assuming a 1000-fold enhancement in cell concentration from bubble-scrubbing (Porco *et al.,* 2017, in this issue), which is readily inside the limits of detection for DHM. Furthermore, a microliter of liquid would provide enough liquid sample in order to obtain multiple hologram sequences, increasing the confidence intervals of detection. Enceladus orbiters and landers can collect samples nondestructively as well as collect far greater sample sizes than possible with a multiflyby Saturn orbiter (Porco *et al.,* 2017, in this issue). In sampling the plumes of Enceladus via plume flyby, however, the samples will be collected on relatively large surface areas of a substrate, possibly at speeds of 5 km/s or greater. Methods will need to be developed for robotic extraction of samples from the capture substrate. Capture of intact bacterial cells will also depend upon capture velocity (Jaramillo-Botero *et al.,* 2012), and design of appropriate capture substrates for maximum recovery of organisms should be performed and tested. Multilayer aerogels have been designed for capture of bacteria in Earth's upper atmosphere (Kawaguchi *et al.,* 2014). It can be expected that cells may be recovered at 1–2 km/s capture speeds (Burchell *et al.,* 2001, 2003; Mann *et al.,* 2004), but greater speeds will require novel capture cells or other methods to decelerate the sample nondestructively. These studies should be performed before choosing a mission design.

We should also consider the possibility that bacterial cells might be concentrated in ice grains in Enceladus' plumes or serve as nucleation sites for the grains. On Earth, formation of bubbles in bodies of water leads to concentration of organics and bacteria up to several thousand–fold (See Porco *et al.,* 2017, in this issue). These processes are essential for nutrient cycling and may play a role in the spread of water-borne infections such as Legionnaire's disease (Walls *et al.,* 2014; Quinn *et al.,* 2015). Laboratory-based sea spray models have been constructed in order to quantify the effect (Prather *et al.,* 2013); similar models could be built for the Ocean Worlds. There are two current end-member models for the eruption environment of Enceladus' plume. One invokes gentle triple-point boiling of water (Schmidt *et al.,* 2008), which is likely to occur at the top of the water column, perhaps a few kilometers below the surface; the other suggests more violent exsolution of volatiles such as CO_2_ (Matson *et al.,* 2012), as well as NH_3_ and CH_4_. The degree to which bacteria and/or organics would be concentrated in such systems remains to be thoroughly studied both theoretically and experimentally.

## 6. Conclusion

The lower limit of bacterial detection of off-axis DHM is 2 orders of magnitude more sensitive than conventional light microscopes with similar magnification and resolution. The quantitative phase imaging associated with the technique assists in discriminating cells from minerals. This robust technology may be easily miniaturized and represents a viable complement to chemical techniques for detection of extant microbial life elsewhere in the Solar System. Capture of a sufficient number of intact cells from the plumes of Enceladus is challenging but may be feasible with the proper mission design.

## Supplementary Material

Supplemental data

Supplemental data

Supplemental data

Supplemental data

Supplemental data

## Abbreviations Used

DHM: digital holographic microscopy, digital holographic microscope
EDS: energy-dispersive X-ray spectroscopy
FOV: field of view
LB: lysogeny broth
SNR: signal-to-noise ratio
